# Statistical Mediation Analysis for Models with a Binary Mediator and a Binary Outcome: the Differences Between Causal and Traditional Mediation Analysis

**DOI:** 10.1007/s11121-021-01308-6

**Published:** 2021-11-16

**Authors:** Judith J. M. Rijnhart, Matthew J. Valente, Heather L. Smyth, David P. MacKinnon

**Affiliations:** 1 Department of Epidemiology and Data Science, Amsterdam UMC, Location VU University Medical Center, Amsterdam Public Health Research Institute, Amsterdam, The Netherlands; 2 Center for Children and Families, Department of Psychology, Florida International University, Miami, FL USA; 3 Department of Psychology, Arizona State University, Tempe, AZ USA

**Keywords:** Mediation analysis, Potential outcomes, Counterfactual, Causal inference, Logistic regression, Binary mediator, Binary outcome

## Abstract

Mediation analysis is an important statistical method in prevention research, as it can be used to determine effective intervention components. Traditional mediation analysis defines direct and indirect effects in terms of linear regression coefficients. It is unclear how these traditional effects are estimated in settings with binary variables. An important recent methodological advancement in the mediation analysis literature is the development of the causal mediation analysis framework. Causal mediation analysis defines causal effects as the difference between two potential outcomes. These definitions can be applied to any mediation model to estimate natural direct and indirect effects, including models with binary variables and an exposure–mediator interaction. This paper aims to clarify the similarities and differences between the causal and traditional effect estimates for mediation models with a binary mediator and a binary outcome. Causal and traditional mediation analyses were applied to an empirical example to demonstrate these similarities and differences. Causal and traditional mediation analysis provided similar controlled direct effect estimates, but different estimates of the natural direct effects, natural indirect effects, and total effect. Traditional mediation analysis methods do not generalize well to mediation models with binary variables, while the natural effect definitions can be applied to any mediation model. Causal mediation analysis is therefore the preferred method for the analysis of mediation models with binary variables.

**Supplementary Information:**

The online version contains supplementary material available at 10.1007/s11121-021-01308-6.

Prevention researchers are not only concerned with the question whether two variables are associated, but also *why* two variables are associated. Mediation analysis is an important tool for identifying the causal processes underlying exposure–outcome effects in both experimental and observational studies (MacKinnon, 2008; VanderWeele, 2015). For example, mediation analysis can be used to investigate substance use as a mediator of the association between immigration generation status and suicide attempts (Peña et al., 2008), or to investigate stress eating as a mediator of the association between depression and obesity (Yu et al., 2016).

An important recent methodological advancement in mediation analysis is the development of causal mediation analysis from a potential outcomes or counterfactual perspective. Causal mediation analysis aims at defining and estimating causal effects, rather than the associational effects from traditional mediation analysis (Holland, 1988; Pearl, 2001). Causal mediation analysis distinguishes between causal effect definitions and causal effect estimation. The direct, indirect, and total effect are defined as the difference between two potential outcomes (Holland, 1986; Pearl, 2001). As a result, the causal effect definitions are general and can also be applied to models with binary mediator and outcome variables, and to models with an exposure–mediator (XM) interaction (Imai et al., 2010; Pearl, 2012; Valeri & Vanderweele, 2013). The causal effect estimation depends on the mediation model and can be either parametric or nonparametric. The generalizability of its effect definitions and the model-specific estimators make causal mediation analysis revolutionary, as it clarifies several ambiguities of traditional mediation analysis, including the conflation of the indirect effect estimate and non-collapsibility for models with a binary outcome (MacKinnon et al., 2007; Rijnhart et al., 2021).

At first glance, causal mediation analysis might seem distinct from traditional mediation analysis. However, for certain mediation models, causal mediation analysis subsumes traditional mediation analysis. For models with continuous mediator and outcome variables, traditional mediation analysis can be used to estimate the natural direct and indirect effects from causal mediation analysis (MacKinnon et al., 2020; Rijnhart et al., 2017). For models with a continuous mediator and a binary outcome, the traditional effects correspond to some, but not all natural effects (Rijnhart et al., 2019, 2021).

When the mediator and outcome are both continuous and in the presence of an XM interaction, recoding of the exposure variable provides traditional indirect estimates similar to the natural indirect effect estimates from causal mediation analysis, and group-mean centering of the mediator variable provides traditional direct effect estimates similar to the natural direct effect estimates from causal mediation analysis (MacKinnon et al., 2020). However, this does not necessarily hold for mediation models with a continuous mediator, a binary outcome, and an XM interaction. For the latter situation, group-mean centering of the mediator variable provides traditional direct effect estimates similar to the controlled direct effect estimated at the average mediator values observed in the control and intervention group, rather than estimates of the natural direct effects (Rijnhart et al., 2021).

Even though causal mediation analysis can be considered a revolutionary method that, in some situations, subsumes traditional mediation analysis, it is not often used by substantive researchers (Lapointe-Shaw et al., 2018; Nguyen et al., 2020; Vo et al., 2020). The uptake of causal mediation analysis is especially low for mediation models with a binary outcome (Vo et al., 2020). A reason for this could be that the literature on causal mediation analysis for models with binary variables is more technical than the literature on causal mediation analysis for models with continuous variables. Vo et al. (2020) suggested that the uptake of causal mediation analysis for binary variables can be enhanced through papers that provide detailed instructions on the application of causal mediation analysis. Explication of causal mediation analysis for models with a binary mediator and binary outcome is a goal of this paper.

The main aim of this paper is to clarify the similarities and differences between causal and traditional effect estimators for mediation models with a binary mediator and a binary outcome. We start with an introduction to traditional mediation analysis. Then, we provide a detailed description of the effect definitions and estimation in causal mediation analysis. After this, an empirical data example is used to illustrate the estimation of causal and traditional effects. Finally, we discuss the implications of the results and future directions.

## Traditional Mediation Analysis

**Fig. 1:**
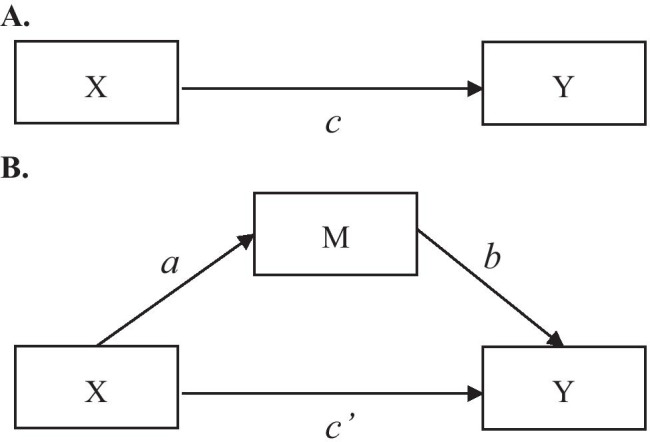
Path diagram of the single mediation model

Mediation analysis decomposes the total exposure-outcome effect (i.e., the *c* path in Fig. 1A) into an indirect effect estimate and a direct effect (MacKinnon, 2008). The indirect effect quantifies the part of the total effect that is transmitted by the mediator (i.e., the *a* and *b* paths in Fig. 1B). The direct effect is the remaining part of the total effect estimate that is not transmitted by the mediator (i.e., the *c’* path in Fig. 1B).

When the mediator and outcome are both binary, the paths in Fig. 1 can be estimated with a sequence of three logistic regression equations (MacKinnon, 2008; MacKinnon et al., 2007):

1 $$logit(Pr\left(Y=1|x\right)) ={i}_{{Y}_{1}}+cX$$

2 $$logit(Pr\left(M=1|x\right)) ={i}_{M}+a\mathrm{X}$$

3 $$logit(Pr\left(Y=1|x, m\right))={i}_{{Y}_{3}}+{c}^{^{\prime}}\mathrm{X}+b\mathrm{M}$$

where in all three equations, *i*_*Y1*_, *i*_*M*_, and *i*_*Y3*_ represent intercept terms. The *c* coefficient in Eq. (1) is the *total effect* of the exposure *X* on the outcome *Y*. The *a* coefficient in Eq. (2) is the effect of the exposure *X* on the mediator *M*. The *c’* coefficient in Eq. (3) is the *direct effect* of the exposure *X* on the outcome *Y*, when adjusted for the mediator *M*, and the *b* coefficient is the effect of the mediator *M* on the outcome *Y*, when adjusted for the exposure *X*. Logistic regression analysis provides effect estimates on the log-odds scale, which can be transformed into odds ratios (ORs) through exponentiation. Any measured confounders can be added to all equations to adjust for confounding.

Traditionally, the *indirect effect* is estimated as either the difference between the total and direct effect, i.e., the difference-in-coefficients method, or as the product of the *a* and *b* coefficients, i.e., the product-of-coefficients method (MacKinnon, 2008; Mackinnon et al., 1995). These two methods are mathematically equivalent when based on linear models (Mackinnon et al., 1995), but not when the outcome is binary and applied to logistic regression coefficients (MacKinnon & Dwyer, 1993; MacKinnon et al., 2007). The difference between the two traditional indirect effect estimates when the outcome is binary is caused by the non-collapsibility of the exposure-outcome effect across mediator values (MacKinnon & Dwyer, 1993; Rijnhart et al., 2021).

Non-collapsibility means that the scale of the logistic regression coefficients depends on the variables in the model (Greenland, 1987; Pang et al., 2016). When variables are added to or omitted from a logistic regression model, the scale of the remaining logistic regression coefficients in the model change. As a result, these coefficients cannot be compared across models. The difference-in-coefficients method conflates the indirect effect and non-collapsibility (MacKinnon & Dwyer, 1993; MacKinnon et al., 2007; Rijnhart et al., 2021). In certain situations, the difference-in-coefficients method can even falsely indicate the presence of an indirect effect (MacKinnon et al., 2007). The product-of-coefficients method does not conflate the indirect effect and non-collapsibility and is therefore preferred for estimating the indirect effect for models with a binary outcome (Rijnhart et al., 2021).

When Eq. (2) is estimated with logistic regression analysis, the *a* coefficient is estimated on the log-odds scale (MacKinnon, 2008; Rijnhart et al., 2019; Smyth, 2019). However, it might be more meaningful to estimate the *a* coefficient with linear regression analysis rather than with logistic regression analysis (Breen et al., 2013; Li et al., 2007; Winship & Mare, 1983). A linear regression model with a binary dependent variable is also referred to as a linear probability model, as it yields effect estimates on the probability scale (Breen et al., 2013; Li et al., 2007; Long, 1997). The purpose of the linear mediator model is to equalize the mediator scale across the *a* and *b* coefficients. In this situation, the outcome model, i.e., Eq. (3), can still be estimated with logistic regression analysis, yielding effect estimates on the log-odds scale.

The *a* coefficient represents the units difference in the mediator produced by a one unit difference in the exposure. To estimate the traditional indirect effect, the *a* coefficient is multiplied with the *b* coefficient, which represents the units difference in the outcome produced by a one unit difference in the mediator (MacKinnon & Dwyer, 1993). The binary mediator variable takes on values of zero or one in the outcome model. The *a* coefficient estimated on the probability scale with a linear probability model also falls within a range between zero and one. When the *a* coefficient is estimated on the log-odds scale with a logistic regression model, it falls within a range between negative infinity and positive infinity (Long, 1997). The linear probability model therefore estimates the *a* coefficient on a mediator scale that corresponds with the zero to one scale of the mediator variable in the outcome model, while logistic regression analysis does not. It therefore makes more sense to multiply the *a* coefficient estimated on the probability scale with the *b* coefficient, than the *a* coefficient estimated on the log-odds scale (Li et al., 2007).

We illustrate the impact of the mediator scale with a small numerical example. Suppose that the observed mediator probability in the intervention group is 0.50, and that the observed mediator probability in the control group is 0.20. This corresponds to a probability difference of 0.30, i.e., 0.50–0.20, and to a log-odds of 1.39, i.e., $ln\left(\frac{0.50/(1-0.50)}{0.20/(1-0.20)}\right)$. Suppose now that the *b* coefficient equals 0.40. The indirect effect based on the *a* coefficient estimated on the probability–difference scale equals 0.12, i.e., 0.30*0.40, while the indirect effect based on the *a* coefficient estimated on the log-odds scale equals 0.56, i.e., 1.39*0.40. As can be seen from this numerical example, the indirect effect based on the log-odds *a* coefficient overestimates the indirect effect based on the probability difference *a* coefficient.

For mediation models with a binary mediator and continuous outcome, the product-of-coefficients and difference-in-coefficients methods provide the same indirect effect estimate when the binary mediator is analyzed using a linear probability model (Li et al., 2007). The mediation analysis is then equivalent to two linear regression models, for which the product-of-coefficients and difference-in-coefficients methods are mathematically equivalent (Mackinnon et al., 1995). However, this equivalence does not hold for models with a binary outcome modelled with logistic regression analysis, as the difference-in-coefficients method is affected by non-collapsibility, while the product-of-coefficients method is not affected by this (MacKinnon et al., 2007; Rijnhart et al., 2019, 2021). The product-of-coefficients method is therefore preferred for models with a binary outcome.

### Exposure–Mediator Interaction

The presence of XM interaction in a mediation model can be assessed by extending Eq. (3) with an XM interaction term (Judd & Kenny, 1981; MacKinnon, 2008):

4 $$logit(Pr\left(Y=1|x, m\right))={i}_{{Y}_{4}}+{c}^{^{\prime}}\mathrm{X}+b\mathrm{M}+hXM$$

where the *h* coefficient represents the effect of the XM interaction on the outcome.

The XM interaction is present when the *h* coefficient is different from zero. The direct and indirect effects estimated based on Eq. (4) are conditional on mediator and exposure values of zero, respectively. In other words, the *c’* coefficient is the direct effect for subjects whose mediator value equals zero, and the indirect effect only holds for subjects whose exposure value equals zero. For a binary intervention variable, a zero value might correspond to the control group. The indirect effect conditional on an exposure value of zero might therefore correspond to the average indirect effect for subjects in the control group (MacKinnon et al., 2020). To derive the mediator–outcome effect estimate (i.e., the* b* coefficient), and thus the indirect effect estimate, for the intervention group, the exposure variable needs to be recoded so that the zero value represents the intervention group.

Even though zero is a meaningful mediator value when the mediator is binary, conditioning the direct effect on a mediator value of zero might not be meaningful in the presence of an XM interaction. Subjects in the control group have a different probability of endorsing the mediator than subjects in the intervention group. For example, for subjects in the control group, the average mediator probability might be 0.60, while for subjects in the intervention group, the average mediator probability might be 0.40. It will be more meaningful to estimate the direct effect conditional on these probabilities than conditional on a mediator value of zero. Group-mean centering can be used to estimate the direct effect conditional on the mediator probabilities observed in the control and intervention groups (MacKinnon et al., 2020). The control-group mean-centered mediator variable is computed by subtracting the average mediator probability in the control group from each subject’s observed mediator value. A mediator value of zero now corresponds to the control-group average mediator probability. When this control-group mean-centered mediator variable is included as M in Eq. (4), the traditional direct effect is conditional on the control-group average mediator probability. The traditional direct effect conditional on the intervention-group average mediator probability can be estimated by centering the mediator variable at the average mediator probability in the intervention-group.

In summary, in traditional mediation analysis, it is more meaningful to estimate the *a* coefficient on the probability difference scale than on the log-odds scale. In the presence of an XM interaction, recoding the exposure variable and group-mean centering the mediator variable provides meaningful estimates of the traditional indirect and direct effects, respectively. In the next section we describe the causal effect definitions and estimation.

## Causal Mediation Analysis

### Causal Effect Definitions

Causal mediation effects are defined in terms of the difference between two potential outcomes (Holland, 1988; Pearl, 2001). A potential outcome is the outcome value that would be observed for a subject, had the subject been exposed to a certain exposure value (Holland, 1986). Suppose that the exposure is a binary intervention variable, where 1 indicates that a subject was assigned to the intervention group, and 0 indicates that a subject was assigned to the control group. In this situation, two potential outcomes can be observed, *Y*_*i*_(1) is the subject’s outcome value when assigned to the intervention group, and *Y*_*i*_(0) is the subject’s outcome value when assigned to the control group. The causal intervention effect is the difference between *Y*_*i*_(1) and *Y*_*i*_(0), i.e., *Y*_*i*_(1) − *Y*_*i*_(0).

To ensure that the difference between *Y*_*i*_(1) and *Y*_*i*_(0) is a causal effect, i.e., attributable to the intervention, the two potential outcomes must be observed simultaneously (Holland, 1986). However, in practice it is not possible to observe two potential outcomes for the same subject at the same time. The inability to observe individual-level causal effects has been referred to as the *fundamental problem of causal inference* (Holland, 1986). Instead of individual-level causal effects, we can estimate average causal effects based on a sample of subjects (Holland, 1986, 1988; Pearl, 2001). Average causal effects are defined as the difference between two average potential outcomes. The average potential outcome in the intervention group is denoted as *E*[*Y*_*i*_(1)], and the average potential outcome in the control group is denoted as *E*[*Y*_*i*_(0)]. Assuming that the intervention and control group are the same with respect to all factors other than intervention assignment, the difference between *E*[*Y*_*i*_(1)] and *E*[*Y*_*i*_(0)] represents the average causal intervention effect, i.e., *E*[*Y*_*i*_(1) − *Y*_*i*_(0)].

The potential outcomes in a mediation model are based not only on exposure values but also on mediator values. This extends the potential outcomes notation to E[Y_i_(1, *m*)] and E[Y_i_(0, *m*)] (Pearl, 2001; Robins & Greenland, 1992). Where E[Y_i_(1, *m*)] is the average potential outcome in the intervention group and under a predetermined mediator value *m*, and E[Y_i_(0, *m*)] is the average potential outcome in the control group and under a predetermined mediator value *m*. Under the assumption that the intervention and control group are the same on all factors other than the intervention assignment, the difference between *E*[*Y*_*i*_(1, *m*)] and *E*[*Y*_*i*_(0, *m*)] is the *controlled direct effect* (CDE), i.e., *E*[*Y*_*i*_(1, *m*) – *Y*_*i*_(0, *m*)]. The CDE is the direct intervention effect when holding the mediator constant at the predetermined value *m* for all subjects (Pearl, 2001; Valeri & Vanderweele, 2013).

Rather than fixing the mediator at a predetermined mediator value, the mediator can also take on the potential value that it would naturally have taken on had the subject been in the intervention or control group (Pearl, 2001). Estimating effects at naturally occurring mediator values in the intervention and control groups provides a way to take into account the XM interaction in the evaluation of direct and indirect effects. A subject’s potential mediator value when assigned to the intervention group is denoted as *M*_*i*_(1), and a subject’s potential mediator value when assigned to the control group is denoted as *M*_*i*_(0). Substituting *m* with these potential mediator values results in four nested potential outcomes: *E*[*Y*_*i*_(0, *M*_*i*_(0))], *E*[*Y*_*i*_(1, *M*_*i*_(0))], *E*[*Y*_*i*_(0, *M*_*i*_(1))], and *E*[*Y*_*i*_(1, *M*_*i*_(1))] (Pearl, 2001; Robins & Greenland, 1992). The differences between these four average nested potential outcomes represent the population-average *natural direct effects*, *natural indirect effects*, and *total effect* when the following four causal assumptions hold (VanderWeele & Vansteelandt, 2009):

1. No unmeasured confounding of the exposure–mediator effect
2. No unmeasured confounding of the exposure–outcome effect
3. No unmeasured confounding of the mediator–outcome effect
4. No confounders of the mediator–outcome effect that are affected by the exposure

The natural direct effects provide insight into the direct effect of the exposure on the outcome, when holding each subject’s mediator constant at its potential value when assigned to either the control or intervention group (Pearl, 2001; Valeri & Vanderweele, 2013). In other words, the natural direct effects are the effects of the intervention on the outcome while blocking the effect through the mediator (Nguyen et al., 2016, 2020). The effect through the mediator is blocked by setting each subject’s mediator to the potential value when either in the control or intervention group, i.e., ${M}_{i}\left(0\right)$ or ${M}_{i}\left(1\right)$, respectively. The *pure natural direct effect* (PNDE) is the difference between two potential outcomes for which the exposure value differs, while holding each subject’s mediator constant at its potential value in the control group, i.e., $E\left[{\mathrm{Y}}_{i}\left(1, {M}_{i}\left(0\right)\right)-{\mathrm{Y}}_{i}\left(0, {M}_{i}\left(0\right)\right)\right]$. In other words, the PNDE is the direct effect of the intervention on the outcome while blocking the effect through the mediator, by setting each subjects’ mediator to ${\mathrm{M}}_{i}\left(0\right)$. The *total natural direct effect* (TNDE) is the difference between two potential outcomes for which the exposure value differs, while holding each subject’s mediator constant at its potential value in the intervention group, i.e., $E\left[{\mathrm{Y}}_{i}\left(1, {M}_{i}\left(1\right)\right)-{\mathrm{Y}}_{i}\left(0, {M}_{i}\left(1\right)\right)\right]$. In other words, the TNDE is the direct effect of the intervention on the outcome while blocking the effect through the mediator, by setting each subjects’ mediator to ${\mathrm{M}}_{i}\left(1\right)$.

The natural indirect effects provide insight into the effect of the exposure on the outcome through the mediator when holding the exposure constant at the control group or intervention group value (Pearl, 2001; Valeri & Vanderweele, 2013). In other words, the natural indirect effects are the effects of the intervention on the outcome through the mediator while blocking the direct intervention effect (Nguyen et al., 2016, 2020). The direct intervention effect is blocked by setting the exposure to either the control or intervention group value, i.e., 0 or 1, respectively. The *pure natural indirect effect* (PNIE) is the difference between two potential outcomes for which each subject’s mediator value differs, while holding the exposure constant at the control-group level, i.e., $E\left[{Y}_{i}\left(0, {M}_{i}\left(1\right)\right)-{Y}_{i}\left(0, {M}_{i}\left(0\right)\right)\right]$. In other words, the PNIE is the indirect effect of the intervention on the outcome through the mediator while blocking the direct intervention effect by setting the exposure to 0. The *total natural indirect effect* (TNIE) is the difference between two potential outcomes for which each subject’s mediator value differs, while holding the exposure constant at the intervention-group level, i.e., $E\left[{Y}_{i}\left(1, {M}_{i}\left(1\right)\right)-{\mathrm{Y}}_{i}\left(1, {M}_{i}\left(0\right)\right)\right]$. In other words, the TNIE is the indirect effect of the intervention on the outcome through the mediator while blocking the direct intervention effect by setting each subject’s exposure to 1. The *total effect* (TE) is the difference between two potential outcomes for which both the intervention and mediator values differ, i.e., $E\left[{Y}_{i}\left(1, {M}_{i}\left(1\right)\right)-{Y}_{i}\left(0, {M}_{i}\left(0\right)\right)\right]$.

The natural effect definitions have two important strengths when compared to the traditional effect definitions. First, whereas the traditional effect definitions are based on linear regression coefficients and therefore depend on parametric assumptions, the natural effect definitions are not dependent on a specific estimation method, and therefore do not depend on parametric assumptions (Holland, 1988; Pearl, 2001). Second, the natural effect definitions incorporate the XM interaction (Pearl, 2001). In other words, the direct and indirect effects are allowed to differ in magnitude across mediator and exposure values, respectively. The traditional effect definitions do not explicitly incorporate the XM interaction. The next section describes how the abovementioned causal effects can be estimated.

### Causal Effect Estimation

Various methods can be used to estimate the average potential outcomes and average natural effects, including a simulation-based approach and a regression-based approach (Hong et al., 2015; Imai et al., 2010; Muthén et al., 2017; Steen et al., 2017; Valeri & Vanderweele, 2013). In the simulation-based approach, the potential mediator values and potential outcome values are simulated for each individual using Eqs. (2) and (4) (Imai et al., 2010). Subsequently, the individual-level estimates of the potential outcomes are subtracted to yield individual-level estimates of the PNDE, TNDE, PNIE, TNIE, and TE. The population-average effect estimates are computed as the averages of these individual-level effect estimates. The simulation-based approach as implemented in the “mediation” R package provides effect estimates on the risk-difference scale (Tingley et al., 2014).

In the regression-based approach, the potential outcomes are estimated based on the estimated coefficients from Eqs. (2) and (4) (Valeri & Vanderweele, 2013). These estimates of the potential outcome values are used to compute the estimates of the PNDE, TNDE, PNIE, TNIE, and TE. Table 1 provides an overview of these effects on the OR scale in terms of regression coefficients (MacKinnon, 2008; VanderWeele, 2015), as implemented in the SAS and SPSS Valeri and Vanderweele (2013) macros and the Stata PARAMED macro (Emsley & Liu, 2013). Note that in the absence of XM interaction, the *h* coefficient equals zero and drops out of the equations. The CDE, PNDE, and TNDE then all reduce to $\mathrm{exp}({c}^{^{\prime}})$, i.e., the natural direct effect (NDE). The PNIE and TNIE then both equal the PNIE in Table 1 and is termed the natural indirect effect (NIE). The TE estimate equals the product of the NDE and NIE estimates in the absence of an XM interaction.

**Table 1:** Overview of the logistic-regression-based causal effects on the odds ratio scale for models with a binary mediator and a binary outcome

Causal effect	Definition	Effect on the odds ratio scale
CDE	$E\left[Y\left(1, m\right)\right]- E\left[Y\left(0,m\right)\right]$	$\mathrm{exp}({c}^{^{\prime}}+hm)$
PNDE	$E\left[Y\left(1,M\left(0\right)\right)\right]-\left[Y\left(0,M(0)\right)\right]$	$\frac{\mathrm{exp}\left({c}^{^{\prime}}\right)(1+\mathrm{exp}\left(b+h+{i}_{M}\right))}{1+\mathrm{exp}(b+{i}_{M})}$
TNDE	$E\left[Y\left(1,M(1)\right)\right]-E\left[Y\left(0,M(1)\right)\right]$	$\frac{\mathrm{exp}\left({c}^{^{\prime}}\right)(1+\mathrm{exp}\left(b+h+{i}_{M}+a\right))}{1+\mathrm{exp}(b+{i}_{M}+a)}$
PNIE	$E\left[Y\left(0,M(1)\right)\right]-E\left[Y\left(0,M(0)\right)\right]$	$\frac{(1+\mathrm{exp}\left({i}_{M}\right))(1+\mathrm{exp}\left(b+{i}_{M}+a\right))}{(1+\mathrm{exp}\left({i}_{M}+a\right))(1+\mathrm{exp}\left(b+{i}_{M}\right))}$
TNIE	$E\left[Y\left(1,M(1)\right)\right]-E\left[Y\left(1,M(0)\right)\right]$	$\frac{(1+\mathrm{exp}\left({i}_{M}\right))(1+\mathrm{exp}\left(b+h+{i}_{M}+a\right))}{(1+\mathrm{exp}\left({i}_{M}+a\right))(1+\mathrm{exp}\left(b+h+{i}_{M}\right))}$
TE	$E\left[Y\left(1,M(1)\right)\right]-E\left[Y\left(0,M(0)\right)\right]$	PNDE*TNIE or TNDE*PNIE

*CDE* controlled direct effect, *PNDE* pure natural direct effect, *TNDE* total natural direct effect, *PNIE* pure natural indirect effect, *TNIE* total natural indirect effect, *TE* total effect

For a complete overview of estimation methods, including their implementation in software programs, we refer to Valente et al. (2020). In the next section, we compare the effect estimates from traditional and causal mediation analysis, using an empirical data example (see the supplemental materials for simulation results comparing traditional and causal mediation analysis). To preserve space, we focus on the regression-based approach in the main paper, as this method provides natural effect estimates on the OR scale and are estimated based on the coefficients from Eqs. 2 and 4. Therefore, this method is closely related to traditional mediation analysis, which also provides effect estimates on the OR scale. The results for the simulation-based approach can be found in the supplementary materials.

## Empirical Data Example

The empirical data example comes from a randomized controlled trial aiming to assess the effectiveness of the Midwestern Prevention Project in the primary prevention of cigarette, alcohol, and marijuana use in adolescents (Pentz et al., 1989). The study was approved by the Institutional Review Board of the University of Southern California. Forty-two schools participated in the study. The data in this example were obtained from eight schools, of which four schools were randomized to receive the intervention. The variables in the study were measured longitudinally. All adolescents in this data example and their parents gave passive written consent for participation in the study. The intervention consisted of ten educational sessions aimed at developing skills to resist drug use. The empirical data example in this paper is based on the example presented in MacKinnon et al. (2007) and investigates the intention to use cigarettes as a mediator of the effect between the intervention and cigarette use. The intention to use cigarettes was measured 2 months after the education program had finished. Cigarette use was measured 3 months after the education program had finished. Only subjects with complete data on the mediator and outcome variable were included in the data example (*n* = 864). Because list-wise deletion was used, substantive conclusions should be approached with caution. Effect estimates based on the mediation analyses were accompanied by 95% percentile bootstrap confidence intervals (CIs) based on 1000 resamples, to take into account the possibly skewed distributions of the effect estimates (Mackinnon et al., 2004; Valeri & Vanderweele, 2013). The empirical data example was analyzed using STATA statistical software release 14.1 (StataCorp, 2016).

## Results

Of the 864 subjects with complete data on the mediator and outcome variables, 493 were in the intervention group, receiving the educational program, and 371 were in the control group (we refer to the supplemental materials for a summary table of the empirical data example). Of the 54 subjects in the intervention group who intended to use cigarettes, 30 subjects ended up using cigarettes (55.6%). Of the 439 subjects in the intervention group who did not intend to use cigarettes, 43 subjects ended up using cigarettes (9.8%). Of the 63 subjects in the control group who intended to use cigarettes, 40 subjects ended up using cigarettes (63.5%). Of the 308 subjects in the control group who did not intend to use cigarettes, 43 subjects ended up using cigarettes (14.0%).

First, we estimated Eqs. (1), (2), (3), and (4) using logistic regression analysis. The exposure–mediator effect (i.e., the *a* coefficient) was additionally estimated with a linear probability model, yielding effect estimates on the probability scale (a complete table with all estimated coefficients is provided in the supplemental materials). Table 2 shows the causal and traditional effect estimates on the OR scale with 95% percentile bootstrap CIs.

**Table 2:** Causal and traditional effect estimates for the empirical data example

Causal mediation analysis				Traditional mediation analysis		
Effect	Estimate (OR)	95% Confidence interval		Effect	Estimate (OR)	95% Confidence interval
Model without exposure–mediator interaction						
CDE/NDE	0.682	0.462 to 1.020		Direct	0.682	0.462 to 1.020
NIE	0.776	0.624 to 0.951		Indirect (linear *a*)	0.865	0.763 to 0.972
				Indirect (logistic *a*)	0.294	0.098 to 0.770
TE	0.529	0.345 to 0.811		Total	0.603	0.427 to 0.854
Model with exposure–mediator interaction						
CDE at M = 0.135	0.676	0.447 to 1.031		Direct at M = 0.135	0.676	0.447 to 1.031
CDE at M = 0.170	0.677	0.451 to 1.019				
CDE at M = 0.110	0.674	0.443 to 1.035				
PNDE	0.703	0.426 to 1.241		Direct (control)	0.677	0.451 to 1.019
TNDE	0.697	0.450 to 1.151		Direct (intervention)	0.674	0.443 to 1.035
PNIE	0.779	0.619 to 0.954		Indirect (control; linear *a*)	0.867	0.759 to 0.973
				Indirect (control; logistic *a*)	0.299	0.094 to 0.731
TNIE	0.772	0.621 to 0.952		Indirect (intervention; linear *a*)	0.863	0.762 to 0.973
				Indirect (intervention; logistic *a*)	0.288	0.096 to 0.796
TE	0.543	0.323 to 0.962		Total	0.603	0.427 to 0.854

*OR* odds ratio, *CDE* controlled direct effect, *NDE* natural direct effect, *NIE* natural indirect effect, *TE* total effect, *PNDE* pure natural direct effect, *TNDE* total natural direct effect, *PNIE* pure natural indirect effect, *TNIE* total natural indirect effect, *M* mediator

When the XM interaction was assumed absent, both causal and traditional mediation analysis provided a direct effect estimate of 0.682. In other words, subjects in the intervention group had a 0.682 times lower odds of using cigarettes 3 months after the educational program finished than subjects in the control group, after adjustment for the intention to use cigarettes. The NIE estimate of 0.776 indicates that subjects in the intervention group had a 0.776 times lower odds of using cigarettes 3 months after the educational program finished than subjects in the control group, through a decrease in the intention to use cigarettes 2 months after the educational program finished. When the *a* coefficient was estimated using a logistic regression model, the traditional indirect effect estimate of 0.294 did not approximate the NIE estimate, but when the *a* coefficient was estimated using a linear probability model, the traditional indirect effect estimate of 0.865 was closer to the NIE estimate of 0.775. The TE estimate of 0.529 indicates that subjects in the intervention group overall had a 0.529 times lower odds of using cigarettes 3 months after the educational program finished than subjects in the control group. The traditional total effect estimate of 0.603 differed from the TE estimate.

When the XM interaction was assumed present, we found that the traditional direct effect estimates were similar to the CDE estimates under the mediator probabilities observed in the control and intervention groups rather than the PNDE and TNDE estimates, respectively. The probability of intending to use cigarettes was 0.170 in the control group (i.e., 63/371) and 0.110 in the intervention group (i.e., 54/493). The CDE based on the control group mediator probability indicates that subjects in the intervention group had a 0.677 times lower odds of using cigarettes 3 months after the educational program finished than subjects in the control group, when each subject’s probability of having the intention to use cigarettes 2 months after the educational program finished was held constant at 0.170. In contrast, the PNDE estimate was 0.703, indicating that subjects in the intervention group had a 0.703 times lower odds of using cigarettes 3 months after the educational program finished than subjects in the control group, when each subject’s intention to use cigarettes 2 months after the educational program finished was held constant at the potential value that would be realized under the control condition. The CDE based on the intervention group mediator probability indicates that subjects in the intervention group had a 0.674 times lower odds of using cigarettes 3 months after the educational program finished than subjects in the control group, when each subject’s probability of having the intention to use cigarettes 2 months after the educational program finished was held constant at 0.110. Here, the TNDE estimate was 0.697, indicating that subjects in the intervention group had a 0.697 times lower odds of using cigarettes 3 months after the educational program finished than subjects in the control group, when each subject’s intention to use cigarettes 2 months after the educational program finished was held constant at the potential value that would be realized under intervention.

The PNDE and TNDE estimates differed from the traditional control-group and intervention-group direct effect estimates, because the PNDE and TNDE are estimated by averaging over the mediator distribution observed in the control and intervention group, respectively, while the traditional control-group and intervention-group direct effect estimates are estimated conditional on the average mediator probability in the control and intervention group, respectively. While effects estimated conditional on the average are the same as the average effect estimate for linear regression models, this does not hold for logistic regression models (VanderWeele, 2009). Therefore, the traditional direct effect estimates are similar to the CDE when estimated conditional on the control and intervention group average mediator probabilities, rather than the PNDE and TNDE estimates.

In our example we estimated the CDE based on the mediator probabilities observed in the control and intervention groups to show that these CDE estimates correspond to the traditional direct effect estimates. In practice, one can estimate the CDE based on any mediator value deemed relevant. For example, one might be interested in the CDE when holding the mediator constant at the average probability in the sample. In our example the average probability was 0.135 (i.e., 117/864). Therefore, the CDE based on this average probability in the sample indicates that subjects in the intervention group had a 0.676 times lower odds of using cigarettes 3 months after the educational program finished than subjects in the control group, when each subject’s intention to use cigarettes was held constant at 0.135.

The PNIE estimate of 0.779 indicates that subjects in the intervention group had a 0.779 times lower odds of using cigarettes 3 months after the educational program finished than subjects in the control group, through a decrease in the intention to use cigarettes 2 months after the educational program finished, when each subject’s exposure was held constant at the control-group level. The TNIE estimate of 0.772 indicates that subjects in the intervention group had a 0.772 times lower odds of using cigarettes 3 months after the educational program finished than subjects in the control group, through a decrease in the intention to use cigarettes 2 months after the educational program finished, when each subject’s exposure was held constant at the intervention-group level. The traditional indirect effect estimates approximated the PNIE and TNIE estimates when the *a* coefficient was estimated using a linear probability model. However, the traditional indirect effect estimates did not approximate the PNIE and TNIE estimates when the *a* coefficient was estimated using a logistic regression model.

The TE estimate of 0.543 indicates that subjects in the intervention group overall had a 0.543 times lower odds of using cigarettes than subjects in the control group. The traditional total effect estimate of 0.603 differed from the TE estimate. Because the *h* coefficient was small in magnitude (i.e., *h* = 0.071), only small differences were observed between the PNDE and TNDE estimates, and the PNIE and TNIE estimates. The PNDE and TNDE estimates were close to the NDE estimate in the mediation model without the XM interaction, and the PNIE and TNIE estimates were close to the NIE estimate. For this data example it would therefore be sufficient to report the results of the mediation analysis without the XM interaction.

## Discussion

The aim of this paper was to clarify the similarities and differences between causal and traditional mediation analysis for mediation models with a binary mediator and a binary outcome. Causal and traditional mediation analysis provided similar direct effect estimates in the absence of an XM interaction, but different indirect and total effect estimates. In the presence of an XM interaction, causal and traditional mediation analysis provided similar estimates of the CDE, but not of the PNDE and TNDE. The traditional indirect effect estimates approximated the PNIE and TNIE estimates when the *a* coefficient was estimated with a linear probability model, but not when the *a* coefficient was estimated with a logistic regression model. The traditional and causal total effect estimates also differed.

The differences between the traditional and natural direct effect estimates in the presence of an XM interaction can be explained through the different types of effects estimated by causal and traditional mediation analysis. Causal mediation analysis provides population-average direct effect estimates while traditional mediation analysis provides conditional direct effect estimates (VanderWeele, 2009). For mediation models with a continuous outcome and an XM interaction, group-mean centered traditional direct effect estimates are similar to the natural direct effect estimates (MacKinnon et al., 2020), because the conditional direct effect estimates based on the group-mean centered mediator variable in linear regression analysis can also be interpreted as the average direct effect in the control and intervention group (VanderWeele, 2009). However, for mediation models with a binary outcome and an XM interaction, the traditional direct effect estimates are similar to CDE estimates in causal mediation analysis, rather than the PNDE and TNDE estimates, because when based on logistic regression, the direct effect estimates conditional on the average mediator probabilities in the control and intervention groups does not correspond to the average direct effect in the control and intervention groups, respectively (Rijnhart et al., 2020). As a result, the traditional direct effect estimates for models with a binary outcome and an XM interaction have the same interpretation as the CDE from causal mediation analysis, rather than the PNDE and TNDE.

The traditional indirect effect estimates approximate natural indirect effect estimates when the *a* coefficient is estimated based on a linear probability model. The causal and traditional estimates were not exactly the same, which is likely because the linear probability model assumes that the exposure–mediator effect is linear, while effects on the probability scale typically follow an S-shape (Long, 1997; Morgan & Teachman, 1988). That is, the effects on the probability scale typically decrease at low and high values of the independent variable. However, the traditional indirect effect estimates include the exposure–mediator effect as a linear effect. The exposure–mediator effect on the probability scale approximates a linear curve when the mediator is common, i.e., when the mediator prevalence approaches 0.50 (Long, 1997). Another drawback of linear probability models is the possibility of predicted values below zero or greater than one. However, it is important to note that unrealistic predicted values are commonly observed for any type of regression with a continuous outcome and is therefore not limited to linear probability models (Long, 1997).

Differences were observed in the causal and traditional total effect estimates. Previous studies demonstrated that for models with a continuous mediator and a binary outcome this difference is caused by the non-collapsibility of the exposure–outcome effect across mediator values (MacKinnon et al., 2007; Rijnhart et al., 2021). For models with a continuous mediator, a binary outcome, and without an XM interaction, the causal and traditional direct and indirect effect estimates are the same, while the total effect estimates differ in magnitude. For models with a binary mediator, binary outcome, and without an XM interaction, the causal and traditional indirect effect estimates differ slightly when the *a* coefficient for the traditional indirect effect is estimated using a linear probability model. Therefore, the differences in the causal and traditional total effect estimates for models with a binary mediator and a binary outcome are likely partly explained by non-collapsibility and partly by the differences in the traditional and natural indirect effects.

To ensure the causal interpretation of the natural effect estimates in practice, it is important to adjust all models for the confounders identified based on the four no-confounding assumptions (Pearl, 2001; Robins & Greenland, 1992; VanderWeele & Vansteelandt, 2009). Adjustment for confounders is even important for intervention studies, as the mediator–outcome effect remains observational and is likely affected by confounders. The natural direct and indirect effect estimates are both biased when confounders of the mediator–outcome effect are ignored. Directed acyclic graphs (DAGs) can be used to help determine which variables are confounders of the effects in the mediation model (Pearl, 2001; Robins, 2003). Various computer programs, such as DAGitty, are available that can be used to create DAGs and to determine the sufficient set of confounders that needs to be adjusted for to ensure a causal interpretation of the effect estimates (Textor et al., 2011). The potential impact of unmeasured confounders can be assessed through sensitivity analyses (Imai et al., 2010; VanderWeele, 2015).

In the main manuscript, we estimated natural effects on the OR scale using the regression-based approach, as this method is most commonly used to analyze binary outcomes (VanderWeele, 2015). However, logistic regression analysis has an important limitation with respect to the estimation of causal mediation effects. ORs only have a causal interpretation when the outcome is rare. When the outcome is rare, the effect estimates on the OR scale approximate risk ratios, which have a population-average interpretation (Greenland, 1987). Therefore, causal mediation analysis poses an additional rare outcome assumption when a logistic regression model is used to estimate causal mediation effects for models with a binary outcome (Vanderweele & Vansteelandt, 2010). This assumption requires the outcome to be rare, i.e., a prevalence of ≤ 0.10, across all strata defined by the exposure and mediator. When the outcome is common, the effect estimates on the OR scale do not have causal interpretations, but the estimates can still be used to test the presence of natural effects (Valeri & Vanderweele, 2013). In this  situation, the simulation-based approach may be used instead of the regression-based approach, as the simulation-based approach provides effect estimates on the risk-difference scale and therefore does not pose a rare outcome assumption (Imai et al., 2010; VanderWeele, 2015).

In previous years, various estimation methods for causal mediation analysis with a binary outcome have been developed and implemented in software (Imai et al., 2010; Muthén et al., 2017; Steen et al., 2017; Valeri & Vanderweele, 2013). This study primarily aimed at comparing the traditional effect estimates with the regression-based causal effect estimates, to provide applied researchers with a better understanding of the similarities and differences between these methods. In the supplementary materials, we showed that the simulation-based approach provides effect estimates on the risk-difference scale, while the commonly used regression-based approach provides effect estimates on the odds ratio scale. Future research should compare the different causal estimation methods to provide insight in the similarities and differences between these methods, including their respective strengths and limitations.

In summary, this study demonstrated that the traditional direct and indirect effects do not generalize well to mediation models with binary variables, as traditional mediation analysis does not provide estimates of the causal mediation effects as defined based on the potential outcomes framework. Causal mediation analysis provides general definitions of causal direct and indirect effects that can be applied to any mediation model, including models with binary variables and models with an XM interaction, to estimate natural direct and indirect effects. Causal mediation analysis is therefore the preferred method for the analysis of mediation models with a binary mediator and a binary outcome.

## Supplementary Information

Below is the link to the electronic supplementary material.

Supplementary file1 (DOCX 519 KB)
