# Supplementary material for: Statistical Mediation Analysis for Models with a Binary Mediator and a Binary Outcome: the Differences Between Causal and Traditional Mediation Analysis
Source: Prev Sci. 2021 Nov 16;24(3):408–18. doi: 10.1007/s11121-021-01308-6 (PMC9108123; doi:10.1007/s11121-021-01308-6)
Supplement: Supplementary file 1 — Supplementary file1 (DOCX 519 KB) [file 11121_2021_1308_MOESM1_ESM.docx]

**Supplemental material for “Statistical mediation analysis for models with a binary mediator and a binary outcome: the differences between causal and traditional mediation analysis”**

Supplementary Appendix 1: Simulation Study

Supplementary Appendix 2: Details of the Empirical Data Example

Supplementary Appendix 3: Estimated Coefficients for the Empirical Data Example

Supplementary Appendix 4: Results for the Simulation-Based Approach

**Supplementary Appendix 1: Simulation Study**

***Simulation Methods***

A Monte Carlo simulation study was designed to assess the similarities and differences between the causal and traditional effect estimates for mediation models with a binary mediator and a binary outcome. The simulated data were generated and analyzed using Stata statistical software release 14.1 (StataCorp, 2016). The exposure, mediator, and outcome were generated from a normal distribution. The parameter values between the continuous exposure, mediator, exposure-mediator (XM) interaction term, and outcome were set to 0, 0.14, 0.39, and 0.59, to approximate zero, small, medium, and large effect sizes, respectively (Cohen, 1988). The exposure and outcome were both split at their respective mean to create binary versions of these variables. The mediator was split at *z*-values corresponding to a prevalence of 0.10, 0.30, and 0.50. Sample sizes were set to 250, 500, 750, and 1 000. The total number of conditions was 3 072 with 1 000 replications per condition, resulting in 3 072 000 observations.

The causal and traditional direct, indirect, and total effects were computed for each observation. For the traditional indirect effect, Equation 2 was estimated using both a logistic regression model and a linear probability model, resulting in two traditional indirect effect estimates for each observation. The differences between the causal and traditional effect estimates were computed, and analysis of variance (ANOVA) was used to assess relevant predictors of these differences. The parameter values, mediator prevalence, and sample size were used as factors. Main effects and interaction effects with a partial eta-squared (*η^2^*) greater than or equal to 0.01 were considered important predictors (Cohen, 1988).

To assess the performance of each effect estimator, the absolute bias, relative bias, mean squared error (MSE), and root mean squared error (RMSE) were computed for each observation (Burton et al., 2006). Absolute bias was computed as the difference between the estimated effect in each simulated observation and the true effect. The absolute bias in the causal and traditional effect estimates were computed with respect to their own true values. That is, the absolute bias in the causal estimators was computed based on the true causal effects, and the absolute bias in the traditional estimators was computed based on the true traditional effects. Relative bias was computed as the ratio of the absolute bias to the true effect. The MSE was computed as the squared absolute bias and is interpretable as a combination of the bias and the variability in the effect estimates. The RMSE was computed as the square root of the MSE and is interpretable as the standard deviations difference between the estimated and true effect.

***Simulation Results***

The logistic regression analyses suffered from convergence issues, especially when the outcome or mediator was rare. We observed non-convergence of the outcome model in 20 observations. These 20 observations were all conditions with a sample size of 250 and a mediator probability of 0.10. For another 13 268 observations we observed that coefficients were omitted from the model due to sparseness of the data. This happened across all sample sizes, but more often in sample sizes of 250 and for large *h* coefficient values. These observations were excluded from the analyses.

Most causal and traditional estimators were unbiased with respect to their own true value and had low variability. Exceptions were the NIE, PNIE, PNDE, and TNDE estimators, which all had a relative bias above 0.10. The relative bias in the NIE and PNIE estimators increased as the mediator prevalence and sample size decreased. The relative bias in the PNDE and TNDE estimators increased as the mediator prevalence and *c’* coefficient decreased in magnitude. In models without XM interaction, the causal and traditional indirect and total effect estimates differed, while the causal and traditional direct effect estimates were the same. In models with XM interaction, all causal and traditional effect estimates differed except for the CDE estimates. The differences between the causal and traditional indirect effect estimates were generally smaller when the *a* coefficient was estimated based on a linear probability model, than when the *a* coefficient was estimated based on a logistic regression model. In the following two sections we describe the factors that explained these differences.

*Models without XM interaction*

Table S1.1 shows the means, absolute bias, relative bias, MSE, and RMSE of the causal and traditional estimators for models without XM interaction. The estimators were generally unbiased with respect to their own true value and showed low variability. An exception was the NIE estimator, which had a relative bias above 0.10. The relative bias in the NIE estimator was a function of the magnitude of the mediator probability (*η^2^* = 0.01), and the two-way interaction between the mediator probability and sample size (*η^2^* = 0.01).

| **Table S1.1** | | | | | |
| --- | --- | --- | --- | --- | --- |
| *Performance measures of causal and traditional estimators for models without exposure-mediator interaction* | | | | | |
| **Effect estimators** | **Mean value** | **Absolute bias** | **Relative bias^a^** | **MSE** | **RMSE** |
| Causal estimators | | | | | |
| CDE/NDE | 0.452 | 0.002 | 0.002 | 0.054 | 0.177 |
| NIE | 0.047 | 0.006 | 0.193 | 0.008 | 0.039 |
| TE | 0.499 | 0.008 | 0.017 | 0.067 | 0.192 |
| Traditional estimators | | | | | |
| Direct effect *c’* | 0.452 | 0.002 | 0.002 | 0.054 | 0.177 |
| Indirect effect *ab*^b^ | 0.245 | 0.023 | 0.079 | 2.316 | 0.289 |
| Indirect effect *ab*^c^ | 0.040 | 0.001 | 0.061 | 0.012 | 0.037 |
| Total effect *c* | 0.482 | <0.001 | -0.001 | 0.035 | 0.143 |
| *Note.* MSE, mean squared error; RMSE, root mean squared error; CDE, controlled direct effect; NDE, natural direct effect; NIE, natural indirect effect; TE, total effect.  ^a^ Relative bias was only calculated when the true effect was larger than zero.  ^b^ Based on a logistic mediator model.  ^c^ Based on a linear mediator model | | | | | |

The difference between the NIE and traditional indirect effect *ab* based on a logistic mediator model was a function of the magnitude of the *a* coefficient (*η^2^* = 0.01), the *b* coefficient (*η^2^* = 0.02), and the two-way interaction between the *a* and *b* coefficients (*η^2^* = 0.01). The difference between the NIE and traditional indirect effect *ab* based on a linear mediator model was a function of the magnitude of the mediator probability (*η^2^* = 0.02), the *a* coefficient (*η^2^* = 0.01), the two-way interaction between the mediator probability and sample size (*η^2^* = 0.01), the two-way interaction between the mediator probability and the *a* coefficient (*η^2^* = 0.02), and the three-way interaction between the mediator probability, sample size, and the *a* coefficient (*η^2^* = 0.01). None of the factors explained the difference between the TE and the traditional total effect. The differences between the causal and traditional total effect estimates are likely caused by a combination of non-collapsibility and the differences between the causal and traditional indirect effect estimates.

Figure S1.1A plots the differences between the causal and traditional indirect effect estimates when the *a* coefficient was estimated based on a logistic regression model. The differences between the causal and traditional indirect effect estimates increased as the *a* and *b* coefficients increased in magnitude. Figure S1.1B plots the differences between the causal and traditional indirect effect estimates when the *a* coefficient was estimated based on a linear probability model. The differences between these two estimates increased as the *a* coefficient increased and the mediator probability decreased in magnitude. The differences between the causal and traditional indirect effect estimates when the *a* coefficient was estimated based on a linear probability model were more pronounced in samples of 250 subjects.

| **Figure S1.1** |
| --- |
| *Average differences between the causal and traditional indirect effect estimates* |
| 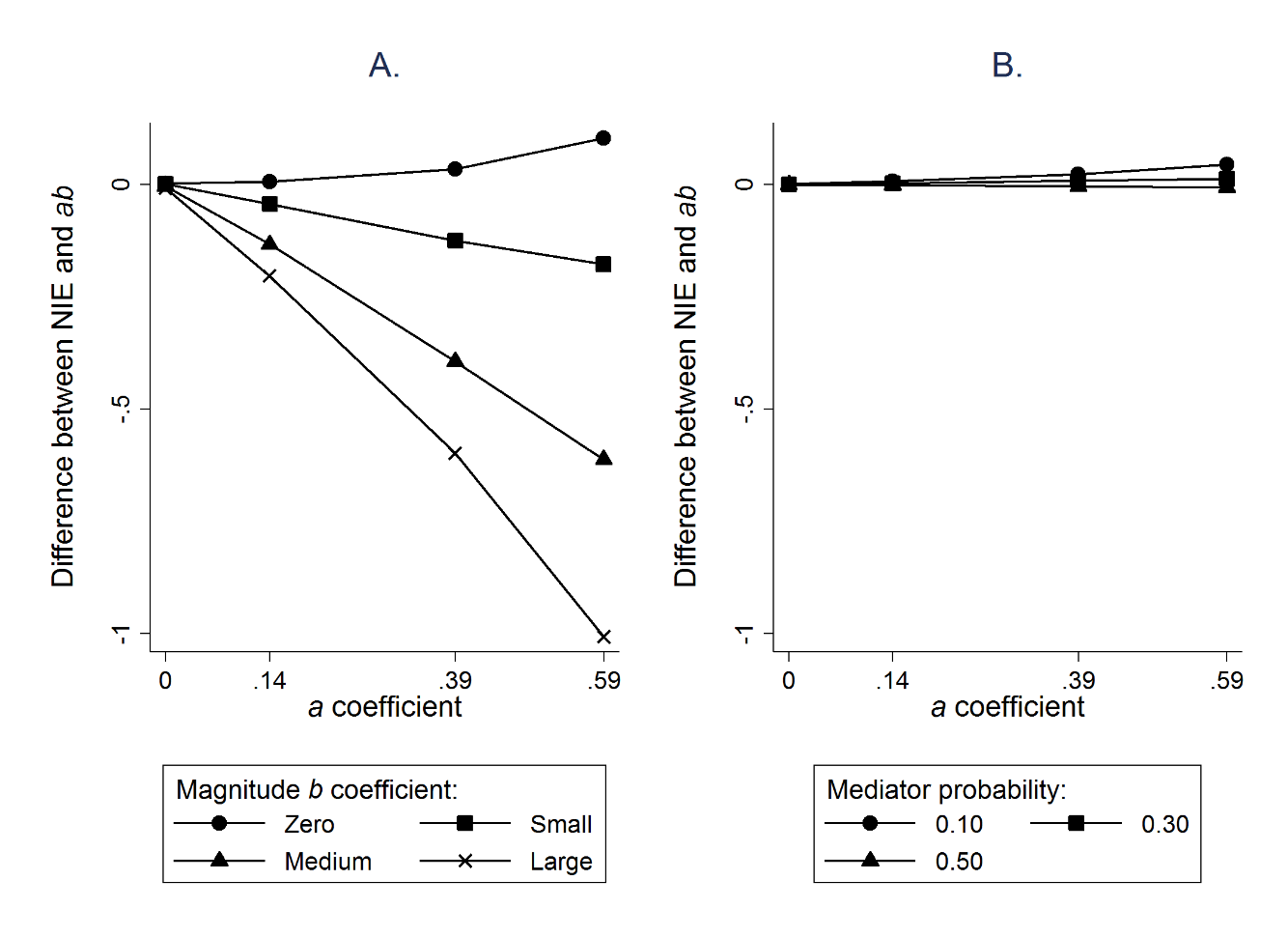 |
| *Note.* Figure S1.1A: the traditional indirect effect estimated based on a logistic mediator model. Figure S1.1B: the traditional indirect effect estimated based on a linear mediator model. |

*Models with XM interaction*

Table S1.2 shows the means and performance measures of the causal and traditional estimators for models with XM interaction. The estimators were generally unbiased with respect to their own true value and showed low variability. Exceptions were the PNDE, TNDE, and PNIE estimators, which all had a relative bias above 0.10. The relative bias in the PNDE estimator was a function of the two-way interaction between the mediator probability and *c’* coefficient (*η^2^* = 0.01) and the three-way interaction between the mediator probability, the *a* coefficient and the *c’* coefficient (*η^2^* = 0.01). None of the factors reached an *η^2^* of 0.01 for explaining the relative bias in the TNDE estimator. The relative bias in the PNIE estimator was a function of the magnitude of the mediator probability (*η^2^* = 0.01), and the two-way interaction between the mediator probability and sample size (*η^2^* = 0.01).

| **Table S1.2** | | | | | |
| --- | --- | --- | --- | --- | --- |
| *Performance measures of causal and traditional estimators for models with exposure-mediator interaction* | | | | | |
| **Effect estimators** | **Mean value^a^** | **Absolute bias** | **Relative bias^a^** | **MSE** | **RMSE** |
| *Causal estimators* | | | | | |
| CDE | 0.593 | 0.003 | -0.012 | 0.043 | 0.152 |
| PNDE | 0.627 | -0.018 | -0.166 | 0.344 | 0.188 |
| TNDE | 0.668 | -0.021 | -0.102 | 0.419 | 0.205 |
| PNIE | 0.047 | 0.005 | 0.182 | 0.008 | 0.038 |
| TNIE | 0.089 | 0.003 | 0.049 | 0.005 | 0.045 |
| TE | 0.716 | -0.015 | -0.084 | 0.346 | 0.196 |
| *Traditional estimators* | | | | | |
| Controlled direct effect | 0.593 | 0.003 | -0.012 | 0.043 | 0.152 |
| Control-group direct effect | 0.573 | 0.003 | -0.010 | 0.039 | 0.150 |
| Intervention-group direct effect | 0.612 | 0.003 | -0.013 | 0.054 | 0.158 |
| Control-group indirect effect^b^ | 0.242 | 0.019 | 0.061 | 2.210 | 0.284 |
| Intervention-group indirect effect^b^ | 0.465 | 0.015 | 0.030 | 0.143 | 0.243 |
| Control-group indirect effect^c^ | 0.040 | 0.001 | 0.043 | 0.011 | 0.036 |
| Intervention-group indirect effect^c^ | 0.079 | 0.001 | 0.017 | 0.003 | 0.038 |
| Total effect *c* | 0.621 | 0.001 | <0.001 | 0.035 | 0.143 |
| *Note.* MSE, mean squared error; RMSE, root mean squared error; CDE, controlled direct effect; PNDE, pure natural direct effect; TNDE, total natural direct effect; PNIE, pure natural indirect effect; TNIE, total natural indirect effect; TE, total effect.  ^a^ Relative bias was only calculated when the true effect was larger than zero.  ^b^ Based on a logistic mediator model.  ^c^ Based on a linear mediator model. | | | | | |

The difference between the PNDE and the traditional control-group direct effect was a function of the magnitude of the mediator probability (*η^2^* = 0.01), the two-way interaction between the mediator probability and sample size (*η^2^* = 0.01), and the two-way interaction between the mediator probability and the *a* coefficient (*η^2^* = 0.01). The difference between the TNDE and the traditional intervention-group direct effect was a function of the magnitude of the mediator probability (*η^2^* = 0.01), the magnitude of the *a* coefficient (*η^2^* = 0.01), the two-way interaction between the mediator probability and sample size (*η^2^* = 0.01), the two-way interaction between the mediator probability and the *a* coefficient (*η^2^* = 0.01), and the three-way interaction between the mediator probability, the *a* coefficient, and sample size (*η^2^* = 0.01).

The difference between the PNIE and the traditional control-group indirect effect based on a logistic mediator model was a function of the magnitude of the *b* coefficient (*η^2^* = 0.01), and the two-way interaction of the *a* and *b* coefficients (*η^2^* = 0.01). The difference between the TNIE and the traditional intervention-group indirect effect based on a logistic mediator model was a function of the magnitude of the mediator probability (*η^2^* = 0.01), the *a* coefficient (*η^2^* = 0.14), the *b* coefficient (*η^2^* = 0.04), the *h* coefficient (*η^2^* = 0.04), the two-way interaction between the mediator probability and the *a* coefficient (*η^2^* = 0.01), the two-way interaction between the *a* and *b* coefficients (*η^2^* = 0.03), and the two-way interaction between the *a* and *h* coefficients (*η^2^* = 0.03).

The difference between the PNIE and the traditional control-group indirect effect based on a linear mediator model was a function of the magnitude of the mediator probability (*η^2^* = 0.02), the *a* coefficient (*η^2^* = 0.01), and the two-way interaction between the mediator probability and the *a* coefficient (*η^2^* = 0.01). The difference between the TNIE and the traditional intervention-group indirect effect based on a linear mediator model was a function of the magnitude of the mediator probability (*η^2^* = 0.09), the *a* coefficient (*η^2^* = 0.02), the two-way interaction between the mediator probability and the *a* coefficient (*η^2^* = 0.06), the two-way interaction between the mediator probability and the *b* coefficient (*η^2^* = 0.03), the two-way interaction between the mediator probability and the *h* coefficient (*η^2^* = 0.03), the three-way interaction between the mediator probability, the *a* coefficient, and the *b* coefficient (*η^2^* = 0.02), and the three-way interaction between the mediator probability, the *a* coefficient, and the *h* coefficient (*η^2^* = 0.02).

The difference between the TE and the traditional total effect was a function of the magnitude of the mediator probability (*η^2^* = 0.01), the *h* coefficient (*η^2^* = 0.01), the two-way interaction between the mediator probability and the *a* coefficient (*η^2^* = 0.01), and the two-way interaction between the mediator probability and sample size (*η^2^* = 0.01).

Figure S1.2A plots the differences between the PNDE estimates and the traditional control-group direct effect estimates as a function of the *a* coefficient and mediator probability. Figure S1.2B plots the differences between the TNDE estimates and the traditional intervention-group direct effect as a function of the *a* coefficient and mediator probability. The differences between the causal and traditional direct effect estimates increased as the *a* coefficient value increased and the mediator probability decreased in magnitude. The relatively large differences between the PNDE and the traditional control-group direct effect estimates for a mediator probability of 0.10 were mainly due to differences observed in samples of 250 subjects. The differences between the causal and traditional indirect effect estimates followed similar patterns as in Figure S1.1 The differences between the TNIE estimates and the traditional intervention-group indirect effect estimates were more pronounced when the *h* coefficient increased in magnitude.

| **Figure S1.2** |
| --- |
| *Average differences between the PNDE and TNDE and the traditional control-group and intervention-group direct effect estimates, respectively* |
| 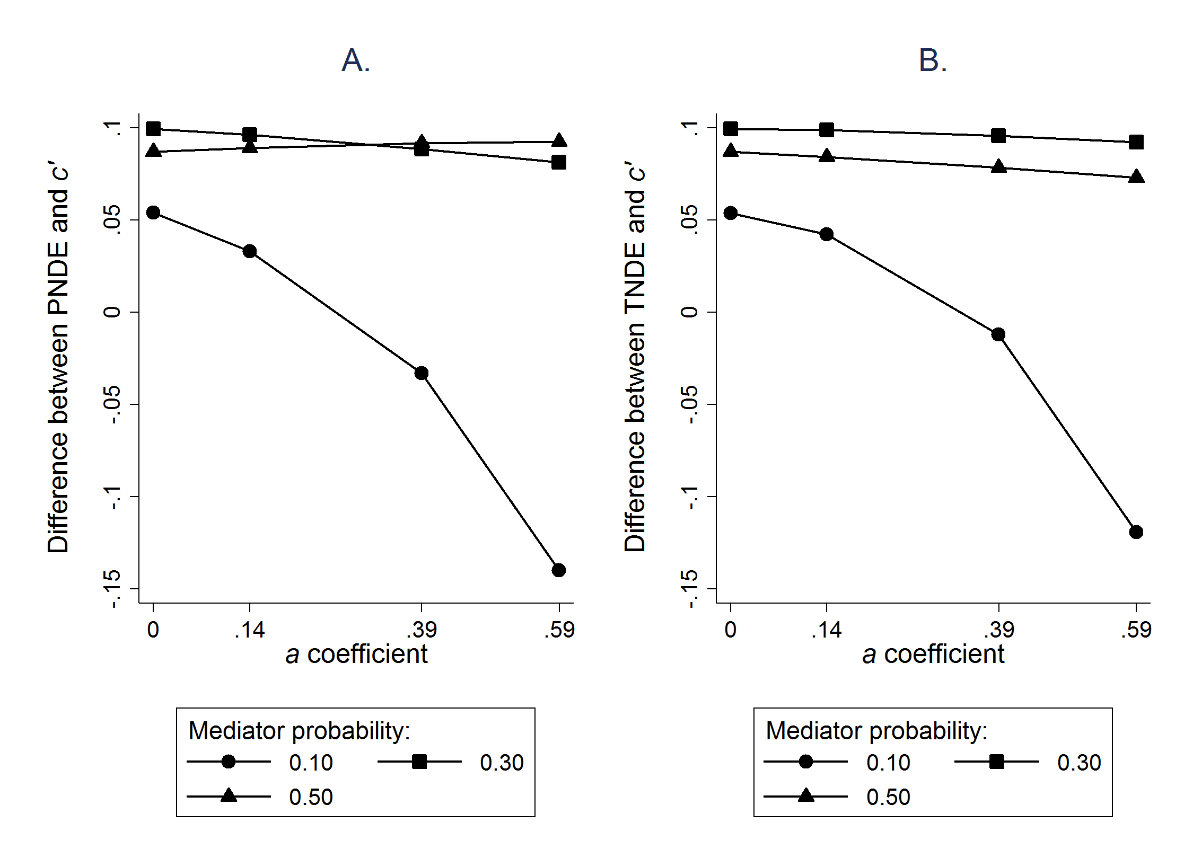 |
| *Note.* Figure S1.2A: differences between the PNDE estimates and the traditional control-group direct effect estimates. Figure S1.2B: differences between the TNDE estimates and the traditional intervention-group direct effect estimates. |

Figure S1.3 plots the differences between the causal and traditional total effect estimates as a function of the *a* coefficient and mediator probability. Figure S1.3A plots these differences based on observations with a zero to small *h* coefficient, and Figure S1.3B plots these differences based on observations with a medium to large *h* coefficient. The differences between the causal and traditional total effect estimates increased as the *h* coefficient increased in magnitude. The relatively large differences between the causal and traditional total effect estimates for a mediator probability of 0.10 were mainly due to differences observed in samples of 250 subjects.

| **Figure S1.3** |
| --- |
| *Average differences between the TE estimate and the traditional total effect c across values of the h coefficient* |
| 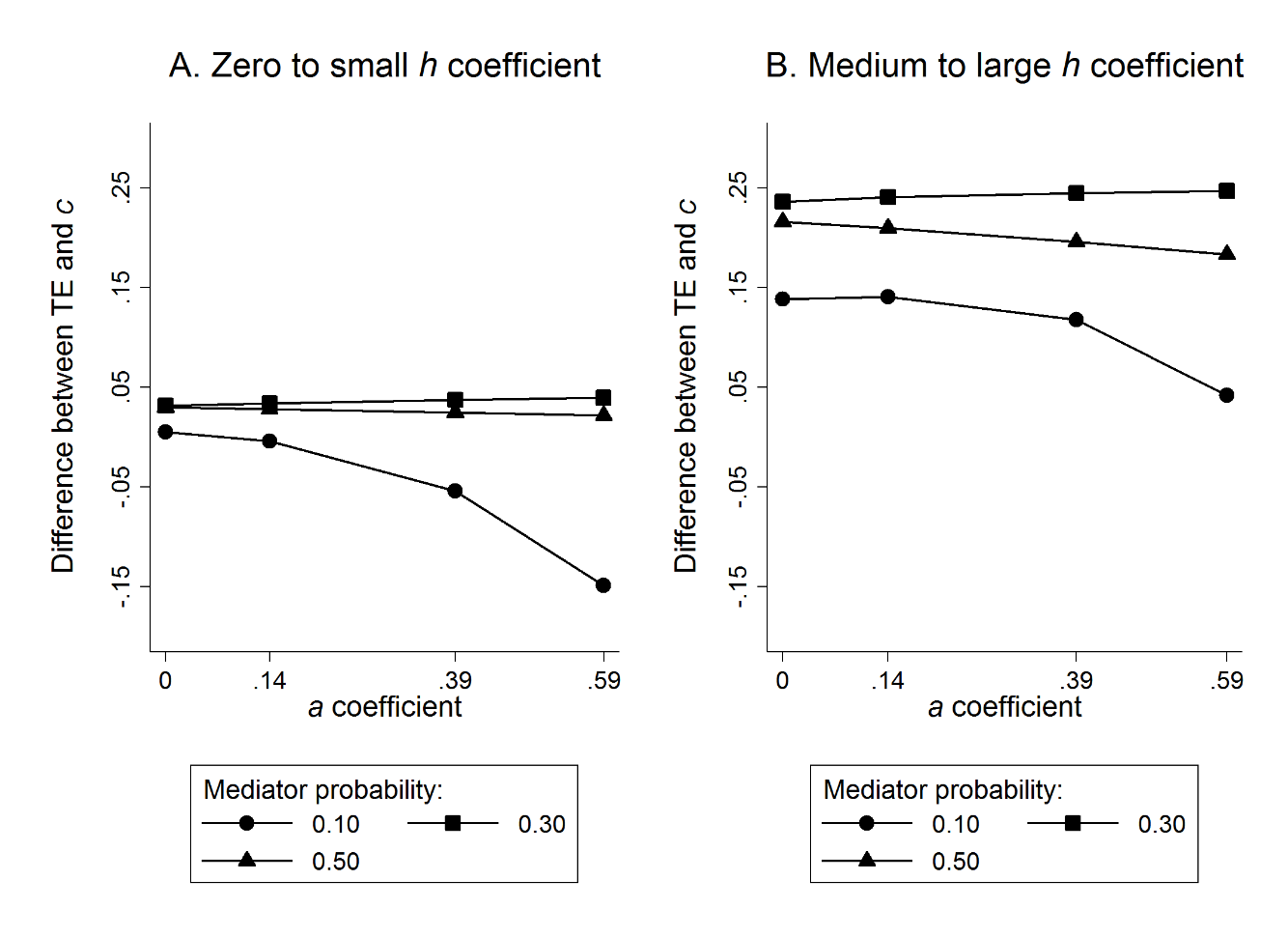 |
| *Note.* Figure S1.3A: based on observations with a zero to small *h* coefficient. Figure S.3B: based on observations with a medium to large *h* coefficient. |

To summarize, the causal natural direct effect estimates and traditional direct effect estimates are similar in the absence of XM interaction, but not in the presence of XM interaction. The causal and traditional indirect and total effect estimates differ for both models with and without XM interaction. The traditional product-of-coefficients estimator approximates the causal NIE estimator when the *a* coefficient is estimated based on a linear probability model, as the exposure-mediator effect in causal mediation analysis is also estimated on the probability scale.

***References for Supplementary Appendix 1***

Burton, A., Altman, D. G., Royston, P., & Holder, R. L. (2006). The design of simulation studies in medical statistics. *Statistics in Medicine*, *25*(24), 4279-4292.

Cohen, J. (1988). *Statistical power analysis for the behavioral sciences.* Hillsdale, NJ: Erlbaum.

StataCorp, L. (2016). STATA Software (version 14.1). *College Station, TX*, *77845*.

**Supplementary Appendix 2: Details of the Empirical Data Example**

Table S2.1 contains information regarding the prevalence of intention to use cigarettes and cigarette use for individuals in the control and intervention groups. The prevalence of intention to use cigarettes in the intervention group was 11% (54/493) and the prevalence of intention to use cigarettes in the control group was 17% (63/371). The prevalence of cigarette use in the intervention group was 15% (73/493) and the prevalence of cigarette use in the control group was 22% (83/371).

| **Table S2.1** | | | | | | |
| --- | --- | --- | --- | --- | --- | --- |
| *Summary of the data from the empirical data example* | | | | | | |
|  | Using cigarettes | |  | Not using cigarettes | | Total |
|  | Intention | No intention |  | Intention | No intention |  |
| Intervention | 30 | 43 |  | 24 | 396 | 493 |
| Control | 40 | 43 |  | 23 | 265 | 371 |
| Total | 70 | 86 |  | 47 | 661 | 864 |

**Supplementary Appendix 3: Estimated Coefficients for the Empirical Data Example**

Table S3.1 contains the estimated regression coefficients from Equations (1) to (4) and from a linear regression version of Equation (2) based on the data from the empirical example.

| **Table S3.1** | | | | |
| --- | --- | --- | --- | --- |
| *Regression results for the empirical data example* | | | | |
| **Coefficient** | **Estimate^a^** | **Standard error** | ***p*-value** | **95% confidence interval^a^** |
| *Logistic regression model 1:* $logit(Pr\left( Y=1 \vert x \right)) =i_{Y_{1}}+cX$ | | | | |
| Intercept $i_{Y_{1}}$ | -1.244 |  |  |  |
| *c* coefficient | -0.506 | 0.178 | 0.004 | -0.854 to -0.157 |
| *Logistic regression model 2:* $logit(Pr\left( M=1 \vert x \right)) =i_{M}+aX$ | | | | |
| Intercept $i_{M}$ | -1.587 |  |  |  |
| *a* coefficient | -0.509 | 0.200 | 0.011 | -0.900 to -0.117 |
| *Linear probability model 2:* $M=i_{M}+aX+\varepsilon_{M}$ | | | | |
| Intercept $i_{M}$ | 0.170 |  |  |  |
| *a* coefficient | -0.060 | 0.023 | 0.010 | -0.106 to -0.014 |
| *Logistic regression model 3:* $logit(Pr\left( Y=1 \vert x, m \right))=i_{Y_{3}}+c^{'}X+bM$ | | | | |
| Intercept $i_{Y_{3}}$ | -1.828 |  |  |  |
| *c’* coefficient | -0.382 | 0.197 | 0.052 | -0.768 to 0.003 |
| *b* coefficient | 2.407 | 0.222 | <0.001 | 1.972 to 2.841 |
| *Logistic regression model 4:* $logit\left( Pr\left( Y=1 \vert x, m \right) \right)=i_{Y_{3}}+c^{'}X+bM+hXM$ | | | | |
| Intercept $i_{Y_{4}}$ | -1.819 |  |  |  |
| *c’* coefficient | -0.402 | 0.230 | 0.080 | -0.852 to 0.049 |
| *b* coefficient | 2.372 | 0.309 | <0.001 | 1.766 to 2.978 |
| *h* coefficient | 0.071 | 0.443 | 0.872 | -0.797 to 0.940 |
| ^a^ Logistic regression estimates are presented on the log-odds scale. | | | | |

Equation (1) yielded a *c* coefficient of -0.506, which corresponds to an OR of 0.603, indicating that subjects in the intervention group had a 0.603 times lower odds of using cigarettes three months after the educational program finished than subjects in the control group. Equation (2) estimated with logistic regression yielded an *a* coefficient of -0.509, which corresponds to an OR of 0.601, indicating that subjects in the intervention group had a 0.601 times lower odds of intending to use cigarettes two months after the educational program finished than subjects in the control group. The exposure-mediator effect estimated with a linear probability model was equal to -0.060, which indicates that the number of subjects that reported to have the intention to smoke two months after the educational program finished is 6% lower in the intervention group than in the control group.

Equation (3) yielded a *c’* coefficient of -0.382, which corresponds to an OR of 0.682, indicating that subjects in the intervention group had a 0.682 times lower odds of using cigarettes three months after the educational program finished than subjects in the control group, after adjustment for the intention to use cigarettes. The *b* coefficient equaled 2.407, which corresponds to an OR of 11.100, indicating that subjects with the intention to use cigarettes two months after the program finished had a 11.100 times higher odds of using cigarettes three months after the educational program finished than subjects without the intention to use cigarettes, after adjustment for program assignment. The *c’* and *b* coefficients changed minimally after adding the XM interaction term to the model, i.e., when estimating equation (4). The *h* coefficient equaled 0.071, which corresponds to an OR of 1.074, which is a relatively small effect and not statistically significant. This was to be expected because no differential effects of intention on cigarette use were hypothesized between the intervention and control group (Pentz et al., 1989). Even though the *h* coefficient was relatively small and not statistically significant, we did estimate the direct, indirect, and total effects incorporating the exposure-mediator interaction for educational purposes.

***References for Supplementary Appendix 3***

Pentz, M. A., Dwyer, J. H., MacKinnon, D. P., Flay, B. R., Hansen, W. B., Wang, E. Y. I., & Johnson, C. A. (1989). A multicommunity trial for primary prevention of adolescent drug abuse: Effects on drug use prevalence. *JAMA*, *261*(22), 3259-3266.

**Supplementary Appendix 4: Results for the Simulation-Based Approach**

We used the ‘mediation’ package in Rstudio statistical software version 3.6.1 to estimate the effects for the empirical data example using the simulation-based estimation approach (Rstudio Inc., 2019; Tingley et al., 2014). Table 4.1 shows the causal effect estimates on the risk-difference scale. In line with the regression-based results, the results from the simulation-based approach indicate that subjects in the intervention group on average have a lower probability of using cigarettes than subjects in the control group. Similarly to the regression-based effect estimates, the direct effect estimates based on the simulation-based approach are also not statistically significant. Therefore, the statistically significant indirect effect estimates indicate that the preventive effect of the intervention is mainly indirect through its influence on the intention to use cigarettes.

| **Table 4.1**  *Causal effect estimates for the empirical data example based on the simulation-based approach* | | |
| --- | --- | --- |
| **Effect** | **Estimate (OR)** | **95% Confidence Interval**^a^ |
| Model without exposure-mediator interaction | | |
| NDE | -0.046 | -0.096 to 0.001 |
| NIE | -0.035 | -0.060 to -0.001 |
| TE | -0.081 | -0.132 to -0.019 |
| Model with exposure-mediator interaction | | |
| PNDE | -0.048 | -0.100 to 0.002 |
| TNDE | -0.045 | -0.094 to 0.001 |
| PNIE | -0.036 | -0.062 to -0.001 |
| TNIE | -0.033 | -0.057 to -0.001 |
| TE | -0.081 | -0.132 to -0.020 |
| *Note.* OR, odds ratio; NDE, natural direct effect; NIE, natural indirect effect; TE, total effect; PNDE, pure natural direct effect; TNDE, total natural direct effect; PNIE, pure natural indirect effect; TNIE, total natural indirect effect.  ^a^ The mediation package outputs the upper limit of the confidence interval with two decimal points only | | |

***References for Supplementary Appendix 4***

Rstudio Inc. (2019). *Rstudio*. In (Version 3.6.1)

Tingley, D., Yamamoto, T., Hirose, K., Keele, L., & Imai, K. (2014). Mediation: R Package for Causal Mediation Analysis. *Journal of Statistical Software*, *59*(5).
